# Supplementary material for: Dispersive determination of nucleon gravitational form factors
Source: Nat Commun. 2025 Jul 30;16:6979. doi: 10.1038/s41467-025-62278-9 (PMC12307625; doi:10.1038/s41467-025-62278-9)
Supplement: Supplementary file 1 — Supplementary Information [file 41467_2025_62278_MOESM1_ESM.pdf]

Supplementary Material for: “Dispersive Determination of Nucleon Gravitational Form Factors”

Xiong-Hui Cao, Feng-Kun Guo, Qu-Zhi Li, and De-Liang Yao

SUPPLEMENTARY INFORMATION

This supplementary material provides detailed methodological documentation and technical specifications to support the reproducibility of the results reported in the main manuscript. The definitions of the gravitational form factors (GFFs) of pions and nucleons are first introduced. Then, the derivation of the unitarity relation of the meson GFFs is given. The Muskhelishvili-Omnès representation for meson GFFs, along with the matching procedure by utilizing chiral perturbation theory (ChPT), is shown explicitly. Finally, the dispersive representation of nucleon GFFs is presented.

Definitions of GFFs

We use the covariant normalization of one-particle state $\langle p' \mid p\rangle = 2p^0(2\pi)^3\delta^3(\mathbf{p}'-\mathbf{p})$, and introduce the combinations of momenta: $P^\mu = p'^\mu + p^\mu$ and $\Delta^\mu = p'^\mu - p^\mu$. In a theory that is invariant under parity, charge conjugation, and time reversal, the total EMT matrix elements $\left\langle p' \middle| \hat{T}^{\mu\nu}(0) \middle| p\right\rangle$ can be expressed in terms of Lorentz structures constructed from P^μ, Δ^μ, and $g^{\mu\nu}$. The constraint $\Delta_\mu \left\langle p' \middle| \hat{T}^{\mu\nu}(0) \middle| p\right\rangle = 0$ must be satisfied, due to the conservation of total energy and momentum. Consequently, only two independent symmetric tensors, $P^\mu P^\nu$ and $\left(\Delta^\mu\Delta^\nu - g^{\mu\nu}\Delta^2\right)$, are possible. Therefore, a spin-0 pion is characterized by two total GFFs, which are defined as [1–3]

$$\left\langle \pi^a(p') \middle| \hat{T}^{\mu\nu}(0) \middle| \pi^b(p)\right\rangle = \frac{\delta^{ab}}{2}\left[A^\pi(t)P^\mu P^\nu + D^\pi(t)\left(\Delta^\mu\Delta^\nu - tg^{\mu\nu}\right)\right], \tag{1}$$

where $t \equiv \Delta^2 < 0$ and $a, b = 1, 2, 3$ are isospin indices. Note that we work in the exact isospin symmetric limit. Then, by crossing, we obtain the definition of the timelike GFFs from Eq. (1) as

$$\left\langle \pi^a(p')\pi^b(p) \middle| \hat{T}^{\mu\nu}(0) \middle| 0\right\rangle = \frac{\delta^{ab}}{2}\left[A^\pi(t)\Delta^\mu\Delta^\nu + D^\pi(t)\left(P^\mu P^\nu - tg^{\mu\nu}\right)\right], \tag{2}$$

with $t \equiv P^2 > 0$.

Likewise, the total GFFs of a spin-1/2 nucleon are defined as [1, 4, 5]

$$\left\langle N(p') \middle| \hat{T}^{\mu\nu}(0) \middle| N(p)\right\rangle = \frac{1}{4m_N}\bar{u}(p')\left[\hat{A}(t)P^\mu P^\nu + \hat{J}(t)\left(iP^{\{\mu}\sigma^{\nu\}\rho}\Delta_\rho\right) + \hat{D}(t)\left(\Delta^\mu\Delta^\nu - tg^{\mu\nu}\right)\right]u(p)\ , \tag{3}$$

where the normalization of spinors is $\bar{u}(p,s)u(p,s) = 2m_N$. From Eq. (3), we obtain the definition of the timelike GFFs as

$$\left\langle N(p')\bar{N}(p) \middle| \hat{T}^{\mu\nu}(0) \middle| 0\right\rangle = \frac{1}{4m_N}\bar{u}(p')\left[\hat{A}(t)\Delta^\mu\Delta^\nu + \hat{J}(t)\left(i\Delta^{\{\mu}\sigma^{\nu\}\rho}P_\rho\right) + \hat{D}(t)\left(P^\mu P^\nu - tg^{\mu\nu}\right)\right]u(p)\ . \tag{4}$$

The isospin of a nucleon is $I = 1/2$. For a dispersive analysis of GFFs, it is convenient to work in the isospin basis and to decompose the GFFs into isoscalar (“s”) and isovector (“v”) components,

$$\hat{X} = X^s\mathbb{1} + X^v\tau^3\ , \quad X = \{A, J, D\}\ . \tag{5}$$

The isoscalar and isovector GFFs, X^s and X^v, are related to the physical ones, X^p and X^n, via

$$\begin{cases} X^s = \frac{1}{2}\left(X^p + X^n\right), \\ X^v = \frac{1}{2}\left(X^p - X^n\right), \end{cases} \quad \text{or} \quad \begin{cases} X^p = X^s + X^v, \\ X^n = X^s - X^v. \end{cases} \tag{6}$$

In the isospin limit, the QCD EMT is an isoscalar and only the isoscalar components remain. We neglect isospin breaking throughout our work and have $X^N \equiv X^p = X^n = X^s$.

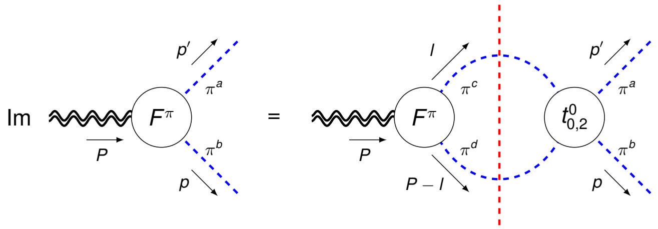

Supplementary Fig. 1. **Elastic unitarity relation for the pion GFFs $F^\pi = \{A^\pi, D^\pi\}$.** The blue dashed lines denote pions, the double wiggly lines represent the external QCD EMT current, and the red vertical dashed line indicates that the intermediate pion pair are to be taken on-shell.

Unitarity and spectral function of pion GFFs

The imaginary parts of the pion GFFs are obtained by inserting a complete set of intermediate states. In the region $t_\pi < t < 16m_\pi^2$, only the $\pi\pi$ intermediate states contribute to the discontinuity (spectral function) of the GFFs, where we use the notation $t_i = 4m_i^2$ $(i = \pi, K, N)$ for the thresholds. In this situation, the spectral function can be computed using the elastic unitarity condition via Cutkosky cutting rule [6], as shown in Supplementary Fig. 1. The discontinuity reads

$$\begin{aligned}
&\text{Disc}\left\langle \pi^a(p')\pi^b(p)\left|\hat{T}^{\mu\nu}(0)\right|0\right\rangle = \frac{\delta^{ab}}{2}\left[\text{Disc}\,A^\pi(t)\Delta^\mu\Delta^\nu + \text{Disc}\,D^\pi(t)\left(P^\mu P^\nu - tg^{\mu\nu}\right)\right]\\
&= \frac{1}{2}\frac{i}{(4\pi)^2}\frac{p_\pi}{\sqrt{t}}\int \mathrm{d}\Omega_l \left\langle \pi^a(p')\pi^b(p)\right|\pi^c(l)\pi^d(P-l)\rangle\left\langle \pi^c(l)\pi^d(P-l)\left|\hat{T}^{\mu\nu}(0)\right|0\right\rangle^*\\
&= \frac{1}{2}\frac{i}{(4\pi)^2}\frac{p_\pi}{\sqrt{t}}\int \mathrm{d}\Omega_l \left(A(t,s,u)\delta^{ab}\delta^{cd} + A(s,t,u)\delta^{ac}\delta^{bd} + A(u,s,t)\delta^{ad}\delta^{bc}\right)\\
&\quad\times\frac{\delta^{cd}}{2}\left[(A^\pi(t))^*(2l-P)^\mu(2l-P)^\nu + (D^\pi(t))^*\left(P^\mu P^\nu - tg^{\mu\nu}\right)\right]\\
&= \frac{1}{2}\frac{i}{(4\pi)^2}\frac{p_\pi}{\sqrt{t}}\int \mathrm{d}\Omega_l\frac{\delta^{ab}}{2}\left[3A(t,s,u)+A(s,t,u)+A(u,s,t)\right]\left[(A^\pi(t))^*(2l-P)^\mu(2l-P)^\nu + (D^\pi(t))^*\left(P^\mu P^\nu - tg^{\mu\nu}\right)\right]\\
&= \frac{1}{2}\frac{i}{(4\pi)^2}\frac{p_\pi}{\sqrt{t}}\frac{\delta^{ab}}{2}\int \mathrm{d}\Omega_l A^{I=0}(t,s,u)\left[(A^\pi(t))^*(2l-P)^\mu(2l-P)^\nu + (D^\pi(t))^*\left(P^\mu P^\nu - tg^{\mu\nu}\right)\right],
\end{aligned}\tag{7}$$

where Ω_l is the solid angle of the integration momentum, and the usual Mandelstam variables are defined as $t = P^2, s = (p'-l)^2$, and $u = (p-l)^2$. The $\pi\pi$ scattering amplitudes (in isospin basis) are represented by $A(t,s,u)$, $A(s,t,u)$ and $A(u,s,t)$. The function $A^{I=0}(t,s,u)$ denotes $\pi\pi$ amplitude with definite isospin $I = 0$, and will be written as $A^{I=0}(t,s)$ for brevity.

Firstly, we calculate the tensor integral $\int \mathrm{d}\Omega_l A^{I=0}(t,s)(2l-P)^\mu(2l-P)^\nu$. According to the Lorentz structure, it can be expressed as

$$\int \mathrm{d}\Omega_l A^{I=0}(t,s)(2l-P)^\mu(2l-P)^\nu = A_1\Delta^\mu\Delta^\nu + A_2\left(P^\mu P^\nu - tg^{\mu\nu}\right),\tag{8}$$

with Lorentz scalar functions A_1 and A_2. We can contract both sides of Eq. (8) with $\Delta_\mu\Delta_\nu$ and $g_{\mu\nu}$, respectively, and obtain

$$\int \mathrm{d}\Omega_l A^{I=0}(t,s)\left[(2l-P)\cdot\Delta\right]^2 = A_1\Delta^4 + A_2\left(-t\Delta^2\right),\quad \int \mathrm{d}\Omega_l A^{I=0}(t,s)(2l-P)^2 = A_1\Delta^2 + A_2\left(-3t\right).\tag{9}$$

These equations are Lorentz invariant and thus can be calculated in any reference frame. For simplicity, we will work in the $\pi\pi$ center-of-mass (c.m.) frame, where the four momenta can be written as

$$l^\mu = \left(\frac{\sqrt{t}}{2}, p_\pi\sin\theta, 0, p_\pi\cos\theta\right),\quad (P-l)^\mu = \left(\frac{\sqrt{t}}{2}, -p_\pi\sin\theta, 0, -p_\pi\cos\theta\right),\tag{10}$$

$$p'^\mu = \left(\frac{\sqrt{t}}{2}, 0, 0, p_\pi\right),\quad p^\mu = \left(\frac{\sqrt{t}}{2}, 0, 0, -p_\pi\right),\tag{11}$$

with p_π the magnitude of the pion 3-momentum in the c.m. frame. Thus, Eq. (9) can be reduced to

$$4p_\pi^2 A_1 + tA_2 = 4p_\pi^2 \int \mathrm{d}\Omega_l A^{I=0}(t,s)\cos^2\theta \ , \quad 4p_\pi^2 A_1 + 3tA_2 = 4p_\pi^2 \int \mathrm{d}\Omega_l A^{I=0}(t,s) \ . \tag{12}$$

Using partial-wave expansion of the elastic $\pi\pi$ scattering [7]

$$A^I(t,s) = 32\pi \sum_J (2J+1) P_J(\cos\theta) t_J^I(t) \ , \tag{13}$$

the two integrals become

$$\int \mathrm{d}\Omega_l A^{I=0}(t,s)\cos^2\theta = \frac{128\pi^2}{3}\left(t_0^0(t) + 2t_2^0(t)\right), \quad \int \mathrm{d}\Omega_l A^{I=0}(t,s) = 128\pi^2 t_0^0(t) \ , \tag{14}$$

and Eq. (8) is reduced to

$$\int \mathrm{d}\Omega_l A^{I=0}(t,s)(2l-P)^\mu(2l-P)^\nu = 128\pi^2 t_2^0(t)\Delta^\mu\Delta^\nu + \frac{512\pi^2}{3t}\left[t_0^0(t) - t_2^0(t)\right](P^\mu P^\nu - tg^{\mu\nu}) \, . \tag{15}$$

Secondly, the other integral in Eq. (7) is straightforward,

$$\int \mathrm{d}\Omega_l A^{I=0}(t,s)\,(P^\mu P^\nu - tg^{\mu\nu}) = 128\pi^2 t_0^0(t)\,(P^\mu P^\nu - tg^{\mu\nu}) \, . \tag{16}$$

Finally, the discontinuity of the EMT matrix element can be written as

$$\begin{aligned} \mathrm{Disc}\left\langle \pi^a(p')\pi^b(p)\left|\hat{T}^{\mu\nu}(0)\right|0\right\rangle &= \frac{\delta^{ab}}{2}\left[\mathrm{Disc}\,A^\pi(t)\Delta^\mu\Delta^\nu + \mathrm{Disc}\,D^\pi(t)\,(P^\mu P^\nu - tg^{\mu\nu})\right] \\ = 2i\frac{2p_\pi}{\sqrt{t}}\frac{\delta^{ab}}{2}&\left[(A^\pi(t))^*\left(\frac{4}{3t}p_\pi^2(t_0^0(t) - t_2^0(t))\,(P^\mu P^\nu - tg^{\mu\nu}) + t_2^0(t)\Delta^\mu\Delta^\nu\right) + (D^\pi(t))^*\, t_0^0(t)\,(P^\mu P^\nu - tg^{\mu\nu})\right] . \end{aligned} \tag{17}$$

Therefore, the spectral functions read

$$\mathrm{Im}\,A^\pi(t) = \frac{2p_\pi}{\sqrt{t}}\left(t_2^0(t)\right)^* A^\pi(t) \ , \tag{18}$$

$$\mathrm{Im}\,D^\pi(t) = \frac{2p_\pi}{\sqrt{t}}\left[\frac{4}{3}\frac{p_\pi^2}{t}\left(t_0^0(t) - t_2^0(t)\right)^* A^\pi(t) + \left(t_0^0(t)\right)^* D^\pi(t)\right] . \tag{19}$$

One sees from Eq. (18) that A^π carries the information on the isoscalar $J^{PC} = 2^{++}$ channel, while D^π mixes the isoscalar 0^{++} and 2^{++} contributions according to Eq. (19).

The matrix elements of the symmetric rank-two tensor $\hat{T}^{\mu\nu}$ can also be decomposed into a sum of two separately conserved irreducible tensors corresponding to well-defined J^{PC}, 0^{++} and 2^{++}, as [8]

$$\left\langle \pi^a(p')\pi^b(p)\left|\hat{T}^{\mu\nu}(0)\right|0\right\rangle = \delta^{ab}\left(T_S^{\mu\nu} + T_T^{\mu\nu}\right), \tag{20}$$

where the scalar and tensor parts are

$$T_S^{\mu\nu} = \frac{1}{3}\left(g^{\mu\nu} - \frac{P^\mu P^\nu}{P^2}\right)\Theta^\pi(t) \ , \tag{21}$$

$$T_T^{\mu\nu} = T^{\mu\nu} - \frac{1}{3}\left(g^{\mu\nu} - \frac{P^\mu P^\nu}{P^2}\right)\Theta^\pi(t) = \left[\Delta^\mu\Delta^\nu + \frac{\Delta^2}{3t}(P^\mu P^\nu - tg^{\mu\nu})\right]A^\pi(t) \ . \tag{22}$$

In Eq.(22), the second equality is derived utilizing Eq. (25) below. $T_S^{\mu\nu}$ is related to the trace of the matrix element, while $T_T^{\mu\nu}$ is traceless. The trace FFs $\Theta^\pi(t)$ is defined as

$$\left\langle \pi^a(p')\pi^b(p)\left|\hat{T}^\mu_{\ \mu}(0)\right|0\right\rangle = \delta^{ab}\Theta^\pi(t) \ . \tag{23}$$

On the other hand, the trace part of Eq. (2) is given by

$$\left\langle \pi^a(p')\pi^b(p)\left|\hat{T}^\mu_{\ \mu}(0)\right|0\right\rangle = \frac{\delta^{ab}}{2}\left[\Delta^2 A^\pi(t) - 3P^2 D^\pi(t)\right] = -\frac{\delta^{ab}}{2}\left[4p_\pi^2 A^\pi(t) + 3tD^\pi(t)\right]. \tag{24}$$

The above two equations are identical and we have

$$\Theta^\pi(t) = -\frac{1}{2}\left(4p_\pi^2 A^\pi(t) + 3tD^\pi(t)\right), \tag{25}$$

which is a pure 0^{++} (scalar) GFF. Using Eqs. (18), (19) and (25), the explicit formula of the spectral function $\operatorname{Im}\Theta^\pi$ reads

$$\operatorname{Im}\Theta^\pi(t) = \frac{2p_\pi}{\sqrt{t}}\left(t_0^0(t)\right)^*\Theta^\pi(t)\ . \tag{26}$$

In fact, significant final-state interactions occur in the 0^{++} channel between the $\pi\pi$ and $K\bar{K}$ states, primarily due to the presence of the $f_0(980)$ resonance. Due to the isoscalar constraint, the $K\bar{K}$ intermediate-state contribution has the similar unitarity relation as Eq. (26) modulo a Clebsh-Gordon coefficient $\frac{2}{\sqrt{3}}$. Now, the unitarity condition (26) is promoted to a matrix form as [9]

$$\operatorname{Im}\mathbf{\Theta}(t) = [\mathbf{T}_0^0(t)]^*\,\mathbf{\Sigma}_0^0(t)\,\mathbf{\Theta}(t)\ , \tag{27}$$

where $\mathbf{\Sigma}_0^0(t) \equiv \operatorname{diag}(\sigma_\pi\theta(t-t_\pi), \sigma_K\theta(t-t_K))$ with $\sigma_i(t) \equiv \sqrt{1-4m_i^2/t}$ $(i=\pi,K)$. The scalar GFFs are collected in

$$\mathbf{\Theta}(t) = \begin{pmatrix} \Theta^\pi(t) \\ \frac{2}{\sqrt{3}}\Theta^K(t)\end{pmatrix}, \tag{28}$$

where $\Theta^K(t) = -\frac{1}{2}\left[4p_K^2 A^K(t) + 3tD^K(t)\right]$. The couple-channel $\pi\pi$-$K\bar{K}$ scattering amplitude in $IJ=00$ wave are parametrized in terms of S-matrix parameters (phases and inelasticity),

$$\mathbf{T}_0^0(t) = \begin{pmatrix} \frac{\eta_0^0(t)e^{2i\delta_0^0(t)}-1}{2i\sigma_\pi} & |g_0^0(t)|e^{i\Psi_0^0(t)} \\ |g_0^0(t)|e^{i\Psi_0^0(t)} & \frac{\eta_0^0(t)e^{2i(\Psi_0^0(t)-\delta_0^0(t))}-1}{2i\sigma_K}\end{pmatrix}. \tag{29}$$

Here the inelasticity parameter $\eta_0^0(t)$ can be related to the partial wave $g_0^0(t)$ of $\pi\pi \to K\bar{K}$ via $\eta_0^0(t) = \sqrt{1-4\sigma_\pi\sigma_K|g_0^0(t)|^2\theta\left(t-t_K\right)}$.

Muskhelishvili-Omnès formalism

Let us first focus on the simple single-channel case of A^π. The unitarity identity (18) represents a single-channel Omnès problem, i.e.,

$$\operatorname{disc}A^\pi(t) = 2iA^\pi(t)\theta(t-t_\pi)\sin\delta(t)e^{-i\delta(t)}\ , \tag{30}$$

where $\delta(t) = \delta_2^0(t) \pmod{\pi}$. The dispersion relation with the single-channel discontinuity admits a standard analytic solution, known as the Omnès representation [10]. Notice that the $I=0$ D-wave $\pi\pi$ scattering is dominated by the $f_2(1270)$ resonance, which has a $\sim 15\%$ branching fraction for decays into 4π and $K\bar{K}$ channels [11]. Such channels lead to a nonvanishing inelasticity, whose effect can be accounted for by replacing the $\pi\pi$ phase shift δ_2^0 in the Omnès solution by the phase of the $\pi\pi$ partial wave ϕ_2^0, which is related to δ_2^0 and η_2^0 through $\left|t_2^0\right|e^{i\phi_2^0} = (\eta_2^0 e^{2i\delta_2^0}-1)/(2i\sigma_\pi)$. The effect has been found to be quite moderate [12]. Thus, the solution to Eq.(18) is given in terms of the Omnès function $\Omega_2^0(t)$ as

$$A^\pi(t) = P_2^\pi(t)\,\Omega_2^0(t)\ , \quad \Omega_2^0(t) \equiv \exp\left\{\frac{t}{\pi}\int_{t_\pi}^\infty \frac{\mathrm{d}t'}{t'}\frac{\phi_2^0(t')}{t'-t}\right\}, \tag{31}$$

with $P_2^\pi(t)$ a polynomial.

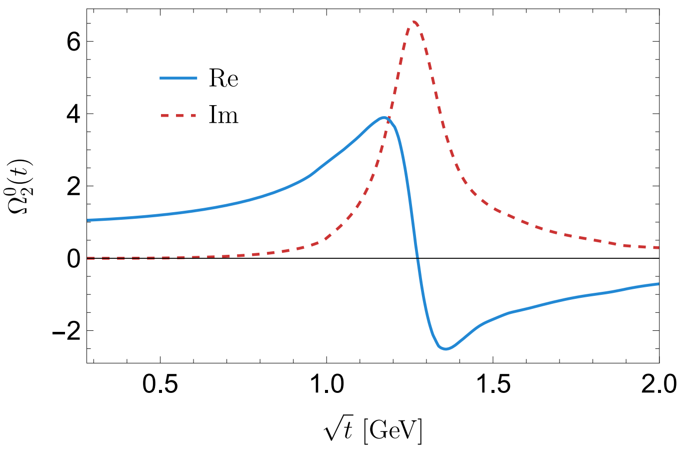

Supplementary Fig. 2. **Results for the real (blue solid) and imaginary (red dashed) parts of the components of the D-wave Omnès function $\Omega_2^0(s)$ up to 2 GeV.**

The D-wave phase shift and inelasticity are taken from the latest crossing symmetric dispersive analysis [13]. We extrapolate the phase shift and inelasticity beyond their endpoints $E_0 \simeq 2$ GeV in the analysis of Ref. [13] via

$$\delta_2^0(t) = \pi + \left(\delta_2^0\left(E_0^2\right) - \pi\right) f_\delta\left(\frac{\sqrt{t}}{E_0}\right), \tag{32}$$

where $f_\delta(x) = 2/\left(1+x^3\right)$ is a smooth extrapolation function connecting the value at the matching point E_0 to the asymptotic value π [14]. A similar extrapolation is used for the inelasticity,

$$\eta_2^0(t) = 1 + \left(\eta_2^0\left(E_0^2\right) - 1\right) f_\eta\left(\frac{\sqrt{t}}{E_0}\right), \tag{33}$$

where f_η has the same form as f_δ.

With the asymptotic behaviors of the phase shift and inelasticity specified, the Omnès integral (31) converges. On the branch cut $t > t_\pi$ of the Omnès function $\Omega_2^0(t)$, one has the issue of Cauchy singularity, which is treated numerically as follows:

$$\begin{aligned}\Omega_2^0(t \pm i\epsilon) &= \exp\left\{\frac{t}{\pi}\int_{t_\pi}^{\infty} \mathrm{d}x \frac{\phi_2^0(x)}{x(x-t\mp i\epsilon)}\right\} = \exp\left\{\frac{t}{\pi}⨍_{t_\pi}^{\infty} \mathrm{d}x \frac{\phi_2^0(x)}{x(x-t)} \pm i\phi_2^0(t)\right\} \\ &= \exp\left\{\frac{t}{\pi}\int_{t_\pi}^{\infty} \mathrm{d}x \frac{\phi_2^0(x)-\phi_2^0(t)}{x(x-t)} + \frac{t}{\pi}\phi_2^0(t)⨍_{t_\pi}^{\infty} \mathrm{d}x \frac{1}{x(x-t)} \pm i\phi_2^0(t)\right\} \\ &= \left|\frac{t_\pi}{(t-t_\pi)}\right|^{\phi_2^0(t)/\pi} \exp\left\{\frac{t}{\pi}⨍_{t_\pi}^{\infty} \mathrm{d}x \frac{\phi_2^0(x)-\phi_2^0(t)}{x(x-t)}\right\} \exp\left\{\pm i\phi_2^0(s)\right\} \\ &\equiv \left|\Omega_2^0(t\pm i\epsilon)\right| \exp\left\{\pm i\phi_2^0(t)\right\}.\end{aligned} \tag{34}$$

The integral representation of the modulus $\left|\Omega_2^0(t\pm i\epsilon)\right|$ is free of Cauchy singularity, which makes it appropriate to numerical integration. The upper integration limit must be chosen sufficiently large to ensure convergence to the target precision. The D-wave Omnès solution is depicted in Supplementary Fig. 2.

As for the polynomial $P_2^\pi(t)$, we use a linear function,

$$P_2^\pi(t) = 1 + \alpha t\,, \tag{35}$$

and the unknown parameter α can be determined by matching to the ChPT result. The $\mathcal{O}(p^4)$, i.e., next-to-leading order (NLO) ChPT result of $A(t)$ reads [2]

$$A^i(t) = 1 - \frac{2L_{12}^r}{F_\pi^2} t\,, \quad i = \pi, K, \eta\,. \tag{36}$$

The LEC L_{12}^r can be estimated by the means of resonance saturation. The tensor meson dominance (TMD) model gives [2]

$$A^\pi(t) \simeq \frac{m_{f_2}^2}{m_{f_2}^2 - t} = 1 + \frac{t}{m_{f_2}^2} + \cdots , \tag{37}$$

with m_{f_2} the mass of the lowest-lying tensor meson $f_2(1270)$, which leads to $L_{12}^r = -\frac{F_\pi^2}{2m_{f_2}^2}$. Matching the Omnès solution in Eq. (31) with the ChPT result up to the linear term in t, we obtain

$$P_2^\pi(t) = 1 + \left(\frac{1}{m_{f_2}^2} - \dot{\Omega}_2^0(0)\right) t = 1 + \left(\frac{1}{m_{f_2}^2} - \frac{1}{\pi}\int_{t_\pi}^\infty \mathrm{d}t' \, \frac{\phi_2^0(t')}{t'^2}\right) t \simeq 1 - (0.01 \text{ GeV}^{-2})t , \tag{38}$$

where we have used the dot notation $\dot{\Omega}_2^0(0) \equiv \frac{\mathrm{d}}{\mathrm{d}t}\Omega_2^0(t)\big|_{t=0}$. Slightly different values have been reported for the $f_2(1270)$ mass at various experiments, e.g., $(1259 \pm 4 \pm 4)$ MeV [15], (1263 ± 12) MeV [16], and (1275 ± 6) MeV [17], among others. The Review of Particle Physics (RPP) provides an averaged value of $m_{f_2} = (1275.4 \pm 0.8)$ MeV [11]. Here, we set $m_{f_2} = (1275 \pm 20)$ MeV to cover all these values as a conservative error estimate.

For the trace GFFs $\Theta^{\pi,K}$, we use the coupled-channel formalism to cover both the S-wave $\pi\pi$ and $K\bar{K}$ channels. The S-wave Omnès matrix is given by the Muskhelishvili-Omnès (MO) solution [10, 18] to the following integral equation

$$\mathbf{\Omega}_0^0(t) = \frac{1}{\pi}\int_{t_\pi}^\infty \frac{\mathrm{d}t'}{t'-t} [\mathbf{T}_0^0(t)]^* \, \mathbf{\Sigma}_0^0(t) \, \mathbf{\Omega}_0^0(t') , \tag{39}$$

with $\mathbf{T}_0^0(t)$ given in Eq. (29). The inputs δ_0^0, Ψ_0^0 and $|g_0^0|$ are taken from Ref. [19] and references therein. An extrapolation similar to Eq. (32) is also conducted for Ψ_0^0,

$$\Psi_0^0(t) = 2\pi + \left(\Psi_0^0\left(E_0^2\right) - 2\pi\right) f_\Psi\left(\frac{\sqrt{t}}{E_0}\right) . \tag{40}$$

where $f_\Psi = f_\delta$. The solution of Eq. (39) is obtained numerically using the discretization procedure described in Ref. [14]. The S-wave Omnès matrix [19] is shown in Supplementary Fig. 3.

The pion and kaon trace GFFs are related to the Omnès matrix by

$$[\mathbf{\Theta}(t)]^T = [\mathbf{P}_0(t)]^T \, \mathbf{\Omega}_0^0(t) , \quad \mathbf{P}_0(t) = \begin{pmatrix} 2m_\pi^2 + \beta_\pi t \\ \frac{2}{\sqrt{3}}\left(2m_K^2 + \beta_K t\right) \end{pmatrix} , \tag{41}$$

with $\mathbf{\Theta}(t)$ defined in Eq. (28). Notice that the polynomial vector $\mathbf{P}_0(t)$ cannot be a constant vector due to the low-energy constraints imposed by chiral symmetry [9]. More explicitly, one has [9]

$$\Theta^\pi(t) = \left(2m_\pi^2 + \beta_\pi t\right) \left(\mathbf{\Omega}_0^0\right)_{11}(t) + \frac{2}{\sqrt{3}}\left(2m_K^2 + \beta_K t\right) \left(\mathbf{\Omega}_0^0\right)_{12}(t) , \tag{42}$$

$$\Theta^K(t) = \frac{\sqrt{3}}{2}\left(2m_\pi^2 + \beta_\pi t\right) \left(\mathbf{\Omega}_0^0\right)_{21}(t) + \left(2m_K^2 + \beta_K t\right) \left(\mathbf{\Omega}_0^0\right)_{22}(t) . \tag{43}$$

The parameters β_π and β_K are related to the slopes at $t = 0$,

$$\begin{aligned} \beta_\pi &= \dot{\Theta}^\pi(0) - 2m_\pi^2 \left(\dot{\mathbf{\Omega}}_0^0\right)_{11}(0) - \frac{4m_K^2}{\sqrt{3}} \left(\dot{\mathbf{\Omega}}_0^0\right)_{12}(0) , \\ \beta_K &= \dot{\Theta}^K(0) - \sqrt{3}m_\pi^2 \left(\dot{\mathbf{\Omega}}_0^0\right)_{21}(0) - 2m_K^2 \left(\dot{\mathbf{\Omega}}_0^0\right)_{22}(0) , \end{aligned} \tag{44}$$

where we have used $\left(\mathbf{\Omega}_0^0\right)_{ij}(0) = \delta_{ij}$, and the slopes $\dot{\Theta}^{\pi,K}(0)$ in ChPT at NLO read [2]

$$\dot{\Theta}^\pi(0) = 1 - 4\left[L_{12}^r + 6(L_{11}^r - L_{13}^r)\right] \frac{m_\pi^2}{F_\pi^2} - \frac{3}{2}\frac{m_\pi^2}{F_\pi^2} I_\pi + \frac{m_\pi^2}{2F_\pi^2} I_\eta = 0.98(2) , \tag{45}$$

$$\dot{\Theta}^K(0) = 1 - 4\left[L_{12}^r + 6(L_{11}^r - L_{13}^r)\right] \frac{m_K^2}{F_\pi^2} - \frac{m_K^2}{F_\pi^2} I_\eta = 0.94(14) , \tag{46}$$

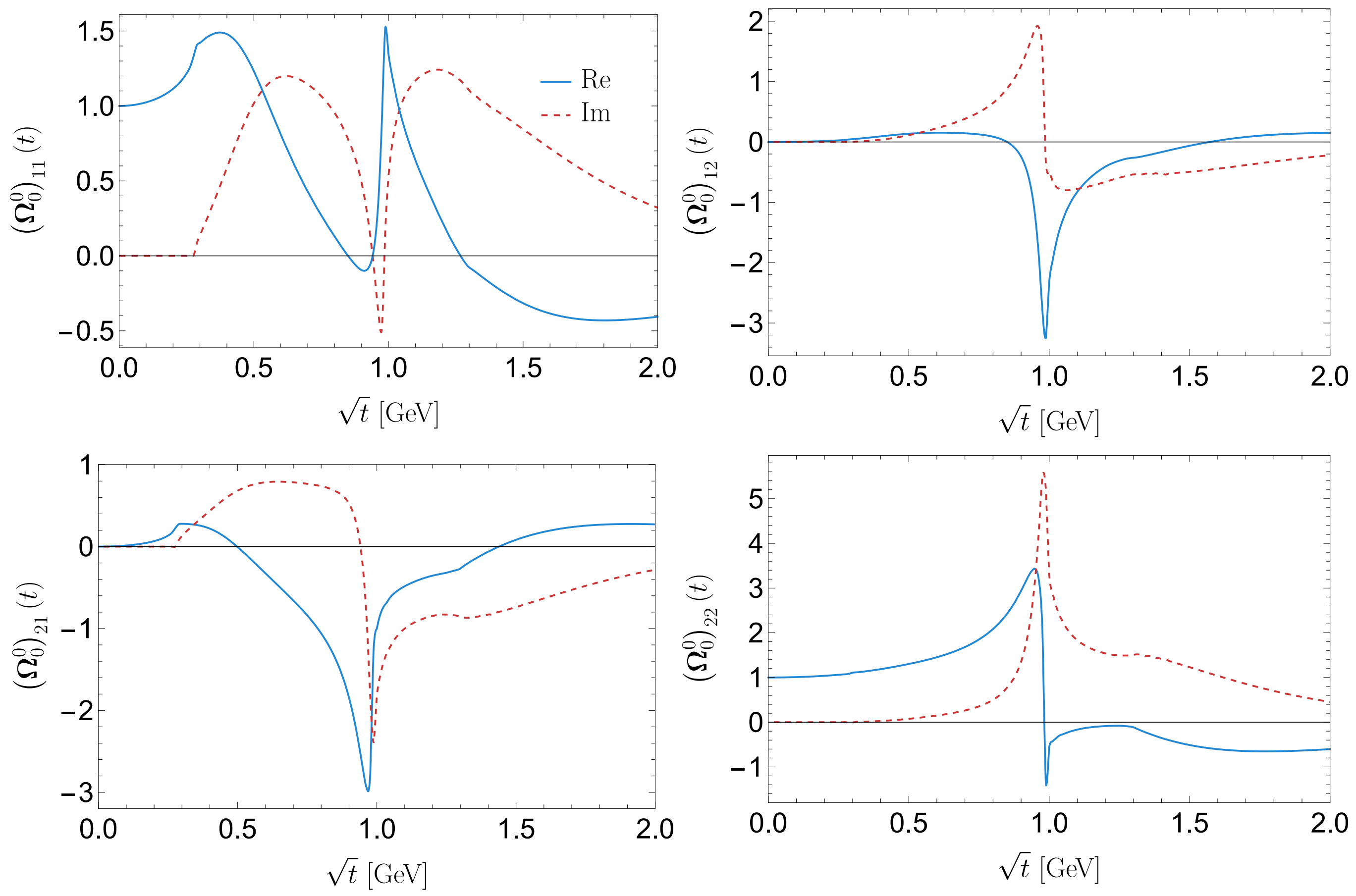

Supplementary Fig. 3. **Results for real (blue solid) and imaginary (red dashed) parts of the components of the *S*-wave $\pi\pi$-$K\bar{K}$ coupled-channel Omnès matrix.**

with the chiral logarithms

$$I_i = \frac{1}{48\pi^2}\left(\ln\left(\frac{\mu^2}{m_i^2}\right) - 1\right). \tag{47}$$

The renormalized low energy constants (LECs) $L_i^r \equiv L_i^r(\mu)$ are scale dependent. In Ref. [2], the values of the LECs were estimated by using dispersion relation techniques and the scalar meson dominance model, as

$$L_{11}^r(\mu = 1\ \text{GeV}) = (1.4 - 1.6) \times 10^{-3}\ , \quad L_{13}^r(\mu = 1\ \text{GeV}) = (0.9 - 1.1) \times 10^{-3}\ . \tag{48}$$

From Eq. (44), one sees that the the inputs from ChPT are only the slopes of the pion and kaon trace GFFs at $t = 0$, which should bear a truncation error due to neglecting higher order chiral corrections. Without having computed these quantities explicitly at the next-to-next-to-leading order (NNLO), the power of effective field theory allows us to estimate the truncation uncertainty to be of $\mathcal{O}(m_\pi^4/\Lambda_\chi^4) = \mathcal{O}(2 \times 10^{-4})$ for the pion case, with $\Lambda_\chi = 4\pi F_\pi$. This is much smaller than the 2% uncertainty of $\dot{\Theta}^\pi(0)$ quoted in Eq. (45). In the kaon case, the NNLO contribution is of order $\mathcal{O}(m_K^4/\Lambda_\chi^4) = \mathcal{O}(3\%)$, again considerably smaller than the 15% uncertainty of $\dot{\Theta}^K(0)$ quoted in Eq. (46)—combining the two errors in quadrature leads to a negligible change. Note that applying such estimates to the NLO contributions would lead to truncation errors of $\mathcal{O}(m_\pi^2/\Lambda_\chi^2) = \mathcal{O}(1.5\%)$ for the pion case and $\mathcal{O}(m_K^2/\Lambda_\chi^2) = \mathcal{O}(18\%)$ for the kaon case if we were to use only the LO results (that is, 1 for both cases). The numerical values in Eqs. (45) and (46) (2% and 6% corrections to the LO results for the pion and kaon cases, respectively) are indeed in line with such estimates.

In addition to the pion GFFs shown in the main text, we also give the results for the kaon trace GFF Θ^K in Supplementary Fig. 4, which is the first dispersive prediction for this quantity.

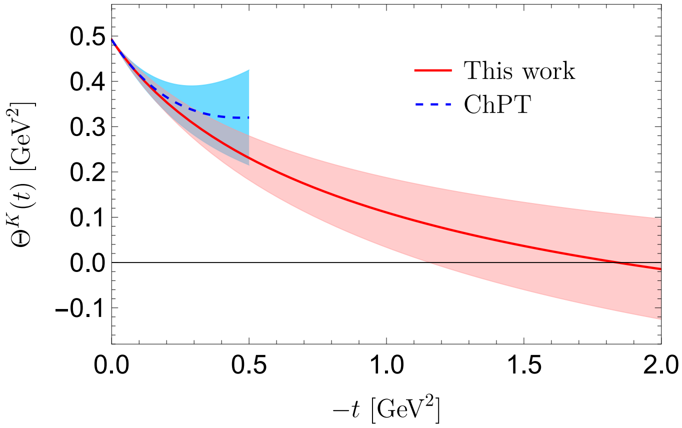

Supplementary Fig. 4. **Trace GFF Θ^K of the kaon.** The blue dashed lines show the NLO ChPT prediction in the small $|t|$ region.

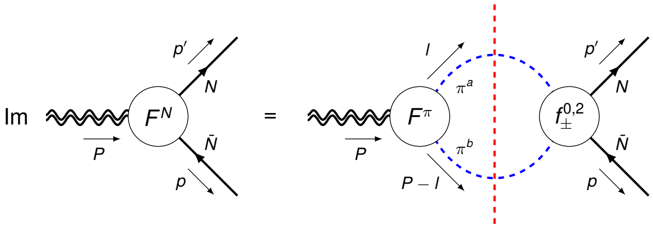

Supplementary Fig. 5. Elastic unitarity relation for the isoscalar nucleon GFFs $F^N = \{A, J, D\}$. The blue dashed, black solid, and double-wiggly lines lines denote pions, nucleons, and the external QCD EMT current, respectively; the red dashed vertical line indicates that the intermediate state $\pi\pi$ are to be taken on-shell.

We have checked that the errors caused by the D-wave Omnès function and the S-wave Omnès matrix are negligible. The uncertainty mainly stems from the SU(3) ChPT estimate of the GFF slopes. The prediction can be compared with future lattice QCD calculations.

Dispersive representation of nucleon GFFs

The discontinuity of the nucleon GFFs can be obtained by inserting a complete set of intermediate states via

$$\text{Disc}\left\langle N(p')\bar{N}(p)\left|\hat{T}^{\mu\nu}(0)\right|0\right\rangle \propto \sum_n \left\langle N(p')\bar{N}(p)\right|n\rangle \left\langle n\left|\hat{T}^{\mu\nu}(0)\right|0\right\rangle^* \delta^4(p+p'-p_n)\ , \tag{49}$$

where $|n\rangle$ denotes asymptotic states with momentum p_n. As for the mesonic case, in the isospin limit, the $I^G\left(J^{PC}\right) = 0^+\left(0^{++}, 2^{++}\right)$ intermediate states $(2\pi, 4\pi, K\bar{K}, \cdots)$ carrying the same quantum numbers as the current $\hat{T}^{\mu\nu}$ contribute.

In the region $t_\pi < t < 16m_\pi^2$, only the $\pi\pi$ intermediate state contributes to the discontinuity of the nucleon GFFs. The discontinuity of the nucleon GFFs can be derived using the Cutkosky cutting rule shown in Supplementary Fig. 5 as

$$\begin{aligned}
&\text{Disc}\left\langle N(p')\bar{N}(p)\left|\hat{T}^{\mu\nu}(0)\right|0\right\rangle \\
&= \frac{1}{4m_N}\bar{u}(p')\left[\text{Disc}\,\hat{A}(t)\Delta^\mu\Delta^\nu + \text{Disc}\,\hat{J}(t)\left(i\Delta^{\{\mu}\sigma^{\nu\}\rho}P_\rho\right) + \text{Disc}\,\hat{D}(t)\left(P^\mu P^\nu - tg^{\mu\nu}\right)\right]v(p) \\
&= \frac{1}{2}\frac{i}{(4\pi)^2}\frac{p_\pi}{\sqrt{t}}\int \mathrm{d}\Omega_l \left\langle N(p')\bar{N}(p)\right|\pi^a(l)\pi^b(P-l)\rangle\left\langle \pi^a(l)\pi^b(P-l)\left|\hat{T}^{\mu\nu}(0)\right|0\right\rangle^* \\
&= \frac{1}{2}\frac{i}{(4\pi)^2}\frac{p_\pi}{\sqrt{t}}\int \mathrm{d}\Omega_l\ \bar{u}(p')\left[\delta^{ab}\mathbb{1}\left(A^+ + \frac{(\not{P}-2\not{l})}{2}B^+\right) + i\epsilon_{bac}\tau^c\left(A^- + \frac{(\not{P}-2\not{l})}{2}B^-\right)\right]v(p)
\end{aligned}$$

$$\times \frac{\delta^{ab}}{2}\left[(A^\pi(t))^*(2l-P)^\mu(2l-P)^\nu+(D^\pi(t))^*(P^\mu P^\nu-tg^{\mu\nu})\right]$$
$$=\frac{1}{2}\frac{i}{(4\pi)^2}\frac{p_\pi}{\sqrt{t}}\int \mathrm{d}\Omega_l\ \bar{u}(p')\frac{3}{2}\left(A^+ + \frac{(\not{P}-2\not{l})}{2}B^+\right)v(p)\left[(A^\pi(t))^*(2l-P)^\mu(2l-P)^\nu+(D^\pi(t))^*(P^\mu P^\nu-tg^{\mu\nu})\right], \tag{50}$$

where $A^\pm$ and $B^\pm$ are Lorentz invariant amplitudes of πN scattering [20], τ^c denotes the Pauli matrices in the isospin space. The Lorentz and isospin decompositions of the elastic πN scattering amplitude can be found in, e.g., Ref. [21].

We need to calculate the following two integrals:

$$\int \mathrm{d}\Omega_l\ A^+(t,s)\ ,\quad \int \mathrm{d}\Omega_l\ B^+(t,s)\frac{(2\not{l}-\not{P})}{2}\ , \tag{51}$$

as well as the tensor ones:

$$\int \mathrm{d}\Omega_l\ A^+(t,s)(2l-P)^\mu(2l-P)^\nu = A_1^+\Delta^\mu\Delta^\nu + A_2^+\left(P^\mu P^\nu - tg^{\mu\nu}\right), \tag{52}$$
$$\int \mathrm{d}\Omega_l\ B^+(t,s)\frac{(2\not{l}-\not{P})}{2}(2l-P)^\mu(2l-P)^\nu = B_1^+\left(2m_N\gamma^{\{\mu}\Delta^{\nu\}}\right)+B_2^+\left(P^\mu P^\nu-tg^{\mu\nu}\right)$$
$$= B_1^+\left(2\Delta^\mu\Delta^\nu + i\Delta^{\{\mu}\sigma^{\nu\}\rho}P_\rho\right)+B_2^+\left(P^\mu P^\nu-tg^{\mu\nu}\right), \tag{53}$$

which have been expressed in terms of Lorentz scalar functions A_1^+, A_2^+, B_1^+ and B_2^+. The Gordon identity $\bar{u}(p')2m_N\gamma^\mu v(p)=\bar{u}(p')\left(\Delta^\mu + i\sigma^{\mu\nu}P_\nu\right)v(p)$ has been used to get the last line.

In the $N\bar{N}$ c.m. frame, the four momenta can be expressed as

$$l^\mu=\left(\frac{\sqrt{t}}{2},p_\pi\sin\theta,0,p_\pi\cos\theta\right),\quad (P-l)^\mu=\left(\frac{\sqrt{t}}{t},-p_\pi\sin\theta,0,-p_\pi\cos\theta\right), \tag{54}$$
$$p'=\left(\frac{\sqrt{t}}{2},0,0,p_N\right),\quad p=\left(\frac{\sqrt{t}}{2},0,0,-p_N\right), \tag{55}$$

with p_N the magnitude of the nucleon three-momentum. Moreover, the on-shell conditions $\bar{u}(p')\not{P}v(p) = \sqrt{t}\bar{u}(p')\gamma^0 v(p)=0$ and $\bar{u}(p')\not{p}'v(p)=m_N\bar{u}(p')v(p)=-p_N\bar{u}(p')\gamma^3 v(p)$ imply

$$\bar{u}(p')\gamma^0 v(p)=0\ ,\quad \bar{u}(p')\gamma^3 v(p) = -\frac{m_N}{p_N}\bar{u}(p')v(p)\ . \tag{56}$$

Contracting both sides of Eq. (52) with $g_{\mu\nu}$ leads to

$$-4p_N^2A_1^+ - 3tA_2^+ = -4p_\pi^2\int \mathrm{d}\Omega_l\ A^+(t,s) = 64\pi^2\left(\frac{p_\pi}{p_N}\right)^2\left(f_+^0(t)-\frac{5m_N}{\sqrt{6}}\left(p_\pi p_N\right)^2 f_-^2(t)\right), \tag{57}$$

where the partial-wave expansion is given by [20, 22]

$$A^I(t,s) = -\frac{8\pi}{p_N^2}\sum_{J=0}^{\infty}\left(J+\frac{1}{2}\right)\left(p_\pi p_N\right)^J\left\{P_J(\cos\theta)f_+^J(t)-\frac{m_N\cos\theta}{\sqrt{J(J+1)}}P'_J(\cos\theta)f_-^J(t)\right\}, \tag{58}$$

with $P'_J(\cos\theta)=\frac{\mathrm{d}P_J(\cos\theta)}{\mathrm{d}\cos\theta}$ and $I=+/-$ if J is even/odd. Here $f_\pm^J(t)$ are the partial-wave $\pi\pi\to N\bar{N}$ scattering amplitudes, and the subscript $+/-$ refers to parallel/antiparallel antinucleon-nucleon helicities such that f_+^0 and $f_\pm^2$ are both isospin even. Furthermore, contracting Eq. (52) with $\Delta^\mu\Delta^\nu$ leads to

$$16p_N^4A_1^+ + 4tp_N^2A_2^+ = 16p_\pi^2p_N^2\int \mathrm{d}\Omega_l\ A^+(t,s)\cos^2\theta\ ,$$
$$= -256\pi^2\left(\frac{p_\pi}{p_N}\right)^2\left[\frac{1}{3}f_+^0(t)+\frac{1}{2}\left(p_\pi p_N\right)^2\left(\frac{4}{3}f_+^2(t)-\sqrt{6}m_Nf_-^2(t)\right)\right]. \tag{59}$$

Therefore, the coefficients of the tensor integral (52) read

$$A_1^+ = 16\pi^2 p_\pi^2 \left(\frac{p_\pi}{p_N}\right)^2 \Gamma^2(t) \ , \tag{60}$$

$$A_2^+ = -\frac{32\pi^2}{t}\left(\frac{p_\pi}{p_N}\right)^2 \left(\frac{2}{3} f_+^0(t) - \frac{2}{3}\left(p_\pi p_N\right)^2 f_+^2(t) - \sqrt{\frac{2}{3}} m_N \left(p_\pi p_N\right)^2 f_-^2(t)\right) , \tag{61}$$

where $\Gamma^2(t) \equiv m_N \sqrt{\frac{2}{3}} f_-^2(t) - f_+^2(t)$.

Contracting Eq. (53) with $g_{\mu\nu}$ and $\Delta^\mu \Delta^\nu$ results in

$$B_1^+ = -\frac{16\pi^2}{\sqrt{6}} \frac{p_\pi^4}{m_N} f_-^2(t) \ , \quad B_2^+ = \frac{64\pi^2}{\sqrt{6}} \frac{m_N p_\pi^4}{t} f_-^2(t) \ , \tag{62}$$

with the partial-wave expansion [20, 22]

$$B^I(t,s) = 8\pi \sum_J \frac{J+\frac{1}{2}}{\sqrt{J(J+1)}} \left(p_\pi p_N\right)^{J-1} P_J'(\cos\theta)\, f_-^J(t) \ . \tag{63}$$

As for the integrals in Eqs. (51), they can be calculated straightforwardly

$$\int \mathrm{d}\Omega_l \ A^+(t,s) = -16\pi^2 \frac{1}{p_N^2}\left(f_+^0(t) - \frac{5m_N}{\sqrt{t}}\left(p_\pi p_N\right)^2 f_-^2(t)\right) , \tag{64}$$

$$\int \mathrm{d}\Omega_l \ B^+(t,s) \frac{(2\not{l} - \not{P})}{2} = \frac{80\pi^2}{\sqrt{6}} m_N p_\pi^2 f_-^2(t) \ . \tag{65}$$

Finally, the spectral functions can be written as,

$$\mathrm{Im}\, A^s(t) = \frac{3p_\pi^5}{\sqrt{6t}}\left[f_-^2(t) + \sqrt{\frac{3}{2}} \frac{m_N}{p_N^2} \Gamma^2(t)\right]^* A^\pi(t) \ , \tag{66}$$

$$\mathrm{Im}\, J^s(t) = \frac{3p_\pi^5}{2\sqrt{6t}}\left(f_-^2(t)\right)^* A^\pi(t) \ , \tag{67}$$

$$\mathrm{Im}\, D^s(t) = -\frac{3m_N p_\pi}{2p_N^2\sqrt{t}}\left[\frac{4p_\pi^2}{3t}\left(\left(f_+^0(t)\right)^* - \left(p_\pi p_N\right)^2 \left(f_+^2(t)\right)^*\right) A^\pi(t) + \left(f_+^0(t)\right)^* D^\pi(t)\right] . \tag{68}$$

Analogously to the above treatment of the pion GFFs, the nucleon matrix elements of $\hat{T}^{\mu\nu}$ can also be decomposed into a sum of two separately conserved tensors corresponding to well-defined J^{PC}, 0^{++} and 2^{++} as

$$\left\langle N(p')\bar{N}(p) \left| \hat{T}^{\mu\nu}(0) \right| 0 \right\rangle = \bar{u}(p')\left(T_S^{\mu\nu} + T_T^{\mu\nu}\right) v(p) \ , \tag{69}$$

where the trace and traceless parts read

$$T_S^{\mu\nu} = \frac{1}{3}\left(g^{\mu\nu} - \frac{P^\mu P^\nu}{P^2}\right)\Theta^s(t) \ , \tag{70}$$

$$\begin{aligned} T_T^{\mu\nu} &= T^{\mu\nu} - \frac{1}{3}\left(g^{\mu\nu} - \frac{P^\mu P^\nu}{P^2}\right)\Theta^s(t) \\ &= \frac{1}{4m_N}\left[\Delta^\mu \Delta^\nu + \frac{\Delta^2}{3t}\left(P^\mu P^\nu - t g^{\mu\nu}\right)\right] A^s(t) + \left[i\Delta^{\{\mu}\sigma^{\nu\}\rho} P_\rho + \frac{2i\sigma^{\rho\kappa}\Delta_\rho P_\kappa}{3t}\left(P^\mu P^\nu - t g^{\mu\nu}\right)\right] J^s(t) \ , \end{aligned} \tag{71}$$

respectively. On the other hand, the trace of Eq. (4) is given by

$$\begin{aligned} \left\langle N(p')\bar{N}(p) \left| \hat{T}^\mu_{\ \mu}(0) \right| 0 \right\rangle &= \bar{u}(p') \frac{1}{4m_N}\left[\Delta^2 A^s(t) + 2i\sigma^{\nu\nu}\Delta_\mu P_\nu J^s(t) - 3tD^s(t)\right] v(p) \\ &= \bar{u}(p') \frac{1}{4m_N}\left[-4p_N^2 A^s(t) + 2tJ^s(t) - 3tD^s(t)\right] v(p) \end{aligned}$$

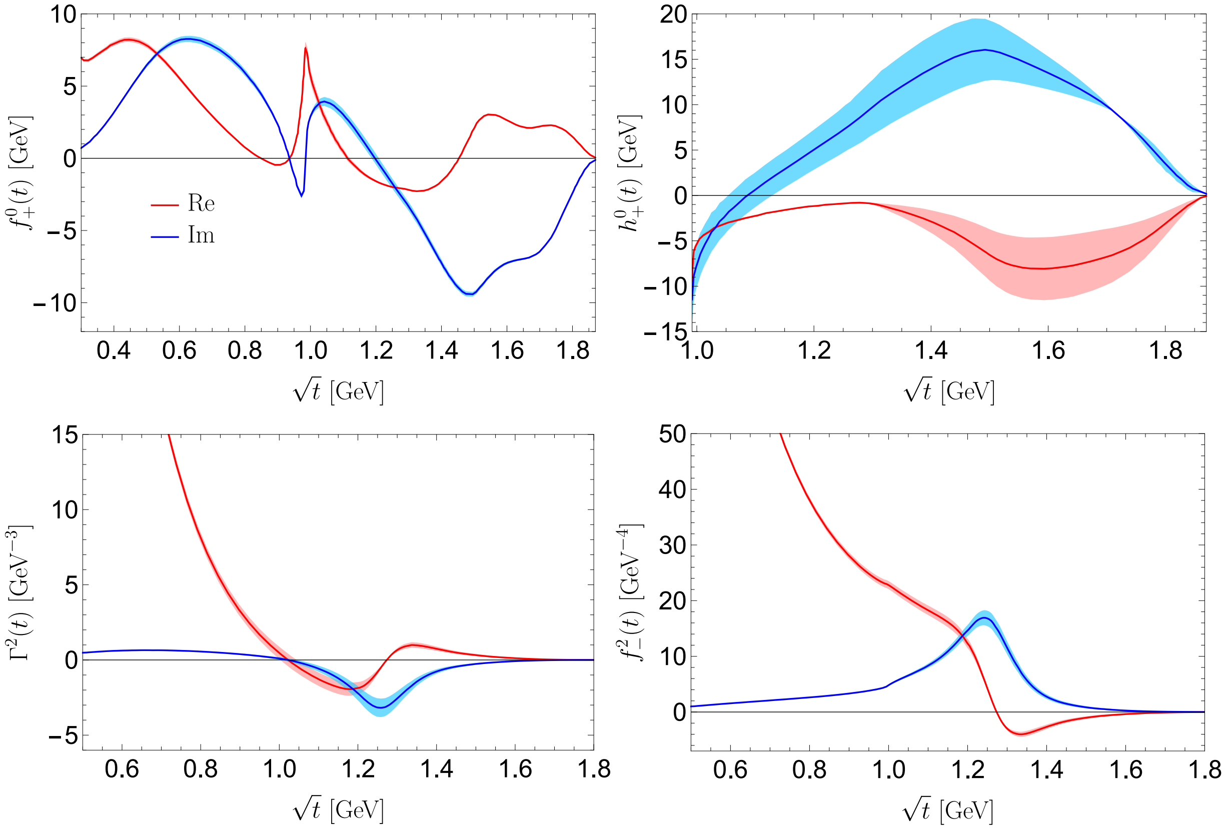

Supplementary Fig. 6. **Results for the $\pi\pi \to N\bar{N}$ partial wave amplitudes f_+^0, Γ^2 and f_-^2 and the $K\bar{K} \to N\bar{N}$ partial wave amplitude h_+^0.**

$$\equiv \bar{u}(p')v(p)\Theta^s(t) \; . \tag{72}$$

Using Eqs. (66), (67), (68) and Eq. (72), the explicit expression of the spectral function $\operatorname{Im}\Theta^s$ can be written as [23]

$$\operatorname{Im}\Theta^s(t) = -\frac{3p_\pi}{4p_N^2\sqrt{t}}\left(f_+^0(t)\right)^*\Theta^\pi(t) \; , \quad \Theta^s(t) = \frac{1}{4m_N}\left[-4p_N^2A^s(t) + 2tJ^s(t) - 3tD^s(t)\right] . \tag{73}$$

It can also be generalized by including the $K\bar{K}$ intermediate state, and the spectral function becomes

$$\operatorname{Im}\Theta^s(t) = -\frac{3}{4p_N^2\sqrt{t}}\left[p_\pi\left(f_+^0(t)\right)^*\Theta^\pi(t)\theta(t-t_\pi) + \frac{4}{3}p_K\left(h_+^0(t)\right)^*\Theta^K(t)\theta(t-t_K)\right] , \tag{74}$$

where h_+^0 is the S-wave isospin-even $K\bar{K} \to N\bar{N}$ scattering amplitude.

The $\pi\pi/K\bar{K} \to N\bar{N}$ S-waves are taken from the rigorous Roy-Steiner equation analyses [12, 19, 24, 25]. This method imposes general constraints on πN scattering amplitudes, such as analyticity, unitarity, and crossing symmetry. The partial waves for $\pi\pi \to N\bar{N}$ are incorporated into a fully crossing-symmetric dispersive analysis, ensuring that the spectral function complies with all analytic S-matrix theory requirements and low-energy data constraints. For the D-wave, as above we adopt an input that is slightly different from that in Ref. [12]. The main difference lies in the fact that we use the phase shift and inelasticity from Ref. [13], which are consistent with the commonly used results up to 1.4 GeV and cover a larger energy range up to around 2 GeV. We have verified that the impact of the D-wave in the t-channel on other partial waves is negligible. Such minor adjustments do not lead to notable changes in the subthreshold parameters in πN scattering, nor do they change the existing results for the S- and P-waves in the s- and t-channels. The results for the $\pi\pi/K\bar{K} \to N\bar{N}$ partial wave amplitudes employed in our analysis are presented in Supplementary Fig. 6.

Once the spectral functions of the nucleon GFFs are determined, the DRs can be immediately formulated for the GFFs. However, identifying the number of subtractions required in the DRs cannot be directly inferred from unitarity

and analyticity alone. The subtraction of the DR relies on the timelike asymptotic behaviour of the GFF. As a simple example, we discuss the asymptotic behavior of A^π, and the conclusion also holds for any GFFs. According to perturbative QCD, $A^\pi(t)$ scales as [26, 27]

$$A^\pi(t) \overset{t\to-\infty}{\sim} \frac{1}{-t} \ , \tag{75}$$

in the asymptotic spacelike region. In the timelike region, unitarity constraint implies that A^π must vanish at infinite momentum transfer $t \to +\infty$ [28]. By applying the Phragmèn-Lindelöf theorem [20, 29], which asserts that the spacelike asymptotic behavior of any FF can be extended to any direction in the complex t plane, we find that A^π scales with the same power in both the spacelike and timelike infinite limits,

$$A^\pi(t) \overset{t\to\pm\infty}{\sim} \frac{1}{|t|} \ . \tag{76}$$

Thus, the GFF A^π allows for an unsubtracted DR. This statement also holds for other GFFs, though the asymptotic behavior depends on the specific GFF [26, 27].

Therefore, the GFFs can be obtained using the unsubtructed DRs,

$$(A, J, \Theta)(t) = \frac{1}{\pi}\int_{t_\pi}^{\infty} \mathrm{d}t' \frac{\mathrm{Im}(A, J, \Theta)(t')}{t'-t} \ . \tag{77}$$

For simplicity, we omit the superscript “s” or “N” for the nucleon GFFs. In practice, the upper limit of the integral is fixed at the two nucleon threshold, t_N. Based on the generic form of the above DRs, it is straightforward to derive sum rules for the normalizations of the nucleon GFFs. We obtain

$$(A, J, \Theta)(0) = \frac{1}{\pi}\int_{t_\pi}^{\infty} \mathrm{d}t' \frac{\mathrm{Im}(A, J, \Theta)\,(t')}{t'} = \left(1, \frac{1}{2}, m_N\right) . \tag{78}$$

By utilizing Eq. (73), one can take the derivative with respect to t and then set $t = 0$,

$$4m_N\Theta'(0) = 4m_N^2 A'(0) - A(0) + 2J(0) - 3D(0) = 4m_N^2 A'(0) - 3D(0) \ . \tag{79}$$

It follows that $D(0)$ is related to a combination of the derivatives of the GFFs A and Θ at $t = 0$,

$$D(0) = \frac{4m_N}{3}\left(m_N A'(0) - \Theta'(0)\right) . \tag{80}$$

Inserting Eq. (78) to the above equation, we can derive the following sum rule

$$D(0) = \frac{4m_N}{3\pi}\int_{t_\pi}^{\infty} \mathrm{d}t' \frac{\mathrm{Im}\left(m_N A(t') - \Theta(t')\right)}{t'^2} \ . \tag{81}$$

Compared to sum rules of normalization, this sum rule converges faster and is less sensitive to the high-energy tails due to the suppression factor $1/t'^2$ rather than $1/t'$ at large t'. It explains why $D(0)$ is more precisely determined than using other methods, such as lattice calculations.

SUPPLEMENTARY REFERENCES

[1] H. Pagels, Energy-Momentum Structure Form Factors of Particles, Phys. Rev. **144**, 1250 (1966).
[2] J. F. Donoghue and H. Leutwyler, Energy and momentum in chiral theories, Z. Phys. C **52**, 343 (1991).
[3] B. Kubis and U.-G. Meißner, Virtual photons in the pion form-factors and the energy momentum tensor, Nucl. Phys. A **671**, 332 (2000), [Erratum: Nucl.Phys.A 692, 647–648 (2001)], arXiv:hep-ph/9908261.
[4] I. Y. Kobzarev and L. B. Okun, Gravitational interaction of fermions, Zh. Eksp. Teor. Fiz. **43**, 1904 (1962).
[5] X.-D. Ji, Gauge-Invariant Decomposition of Nucleon Spin, Phys. Rev. Lett. **78**, 610 (1997), arXiv:hep-ph/9603249.
[6] R. E. Cutkosky, Singularities and discontinuities of Feynman amplitudes, J. Math. Phys. **1**, 429 (1960).
[7] B. Ananthanarayan, G. Colangelo, J. Gasser, and H. Leutwyler, Roy equation analysis of $\pi\pi$ scattering, Phys. Rept. **353**, 207 (2001), arXiv:hep-ph/0005297.
[8] K. Raman, Gravitational form-factors of pseudoscalar mesons, stress-tensor-current commutation relations, and deviations from tensor- and scalar-meson dominance, Phys. Rev. D **4**, 476 (1971).
[9] J. F. Donoghue, J. Gasser, and H. Leutwyler, The Decay of a Light Higgs Boson, Nucl. Phys. B **343**, 341 (1990).

[10] R. Omnes, On the Solution of certain singular integral equations of quantum field theory, Nuovo Cim. **8**, 316 (1958).
[11] S. Navas *et al.* (Particle Data Group), Review of particle physics, Phys. Rev. D **110**, 030001 (2024).
[12] M. Hoferichter, J. Ruiz de Elvira, B. Kubis, and U.-G. Meißner, Roy–Steiner-equation analysis of pion–nucleon scattering, Phys. Rept. **625**, 1 (2016), arXiv:1510.06039 [hep-ph].
[13] P. Bydžovský, R. Kamiński, and V. Nazari, Dispersive analysis of the S-, P-, D-, and F-wave $\pi\pi$ amplitudes, Phys. Rev. D **94**, 116013 (2016), arXiv:1611.10070 [hep-ph].
[14] B. Moussallam, N_f dependence of the quark condensate from a chiral sum rule, Eur. Phys. J. C **14**, 111 (2000), arXiv:hep-ph/9909292.
[15] S. Dobbs, A. Tomaradze, T. Xiao, and K. K. Seth, Comprehensive Study of the Radiative Decays of J/ψ and $\psi(2S)$ to Pseudoscalar Meson Pairs, and Search for Glueballs, Phys. Rev. D **91**, 052006 (2015).
[16] M. Carver *et al.* (CLAS), Photoproduction of the $f_2(1270)$ meson using the CLAS detector, Phys. Rev. Lett. **126**, 082002 (2021), arXiv:2010.16006 [nucl-ex].
[17] E. Klempt, K. V. Nikonov, A. V. Sarantsev, and I. Denisenko, Search for the tensor glueball, Phys. Lett. B **830**, 137171 (2022), arXiv:2205.07239 [hep-ph].
[18] N. I. Muskhelishvili, *Singular Integral Equations: Boundary problems of function theory and their application to mathematical physics* (Springer Dordrecht, 1958).
[19] M. Hoferichter, C. Ditsche, B. Kubis, and U.-G. Meißner, Dispersive analysis of the scalar form factor of the nucleon, JHEP **06**, 063, arXiv:1204.6251 [hep-ph].
[20] G. Höhler, *Pion-Nukleon-Streuung: Methoden und Ergebnisse phänomenologischer Analysen. Teil 2*, in Landolt-Börnstein 9b2, H. Schopper eds., Springer Verlag, Berlin Germany (Springer, 1983).
[21] D.-L. Yao, D. Siemens, V. Bernard, E. Epelbaum, A. M. Gasparyan, J. Gegelia, H. Krebs, and U.-G. Meißner, Pion-nucleon scattering in covariant baryon chiral perturbation theory with explicit Delta resonances, JHEP **05**, 038, arXiv:1603.03638 [hep-ph].
[22] W. R. Frazer and J. R. Fulco, Partial-Wave Dispersion Relations for the Process $\pi\pi \to N + \bar{N}$, Phys. Rev. **117**, 1603 (1960).
[23] M. Hoferichter, J. R. de Elvira, B. Kubis, and U.-G. Meißner, Nucleon resonance parameters from Roy–Steiner equations, Phys. Lett. B **853**, 138698 (2024), arXiv:2312.15015 [hep-ph].
[24] G. E. Hite and F. Steiner, New dispersion relations and their application to partial-wave amplitudes, Nuovo Cim. A **18**, 237 (1973).
[25] X.-H. Cao, Q.-Z. Li, and H.-Q. Zheng, A possible subthreshold pole in S_{11} channel from πN Roy-Steiner equation analyses, JHEP **12**, 073, arXiv:2207.09743 [hep-ph].
[26] X.-B. Tong, J.-P. Ma, and F. Yuan, Gluon gravitational form factors at large momentum transfer, Phys. Lett. B **823**, 136751 (2021), arXiv:2101.02395 [hep-ph].
[27] X.-B. Tong, J.-P. Ma, and F. Yuan, Perturbative calculations of gravitational form factors at large momentum transfer, JHEP **10**, 046, arXiv:2203.13493 [hep-ph].
[28] S. D. Drell and F. Zachariasen, High-energy limit of form factors, Phys. Rev. **119**, 463 (1960).
[29] S. Pacetti, R. Baldini Ferroli, and E. Tomasi-Gustafsson, Proton electromagnetic form factors: Basic notions, present achievements and future perspectives, Phys. Rept. **550-551**, 1 (2015).
